# Evaluating Peer Review of Palliative Radiation Plans at a Canadian Tertiary Care Cancer Center

**DOI:** 10.7759/cureus.57839

**Published:** 2024-04-08

**Authors:** Stephanie Gulstene, Adam Mutsaers, Melissa O'Neil, Andrew Warner, George Rodrigues

**Affiliations:** 1 Division of Radiation Oncology, London Regional Cancer Program, London, CAN; 2 Schulich School of Medicine & Dentistry, Western University, London, CAN

**Keywords:** methods for quality assurance, peer review, palliative radiation therapy, quality assurance (qa), quality assurance and radiation safety

## Abstract

Introduction: Peer review (PR) of palliative-intent radiation plans is an important but understudied component of quality assurance. This retrospective review aims to improve our understanding of palliative PR by examining the characteristics of reviewed plans and peer feedback along with the associated time burden of two different types of PR processes.

Methods: This single-institution, quality assurance project assessed palliative PR between 2018 and 2020. Initially, the PR involved a multi-disciplinary team PR. Subsequently, it transitioned to independent PR by a single physician. Characteristics of reviewed plans and feedback on PR were captured and abstracted. Time requirements of PR were based on self-reported estimates and attendance records.

Results: A total of 1942 cases were reviewed, representing 85.7% (1942/2266) of all palliative-intent plans between 2018 and 2020. A total of 41.1% (n=799) were simple (2D/3D) radiation plans while 56.0% (n=1087) were complex (volumetric modulated arc therapy (VMAT) or tomotherapy) plans. Approximately one-third (30.4%, n=590) of all plans were stereotactic treatments. The rate of any peer feedback was 2.3% (n=45), while the rate of a specific recommended or implemented change was 1.2% (n=24) and 0.9% (n=18), respectively. PR before the start of treatment was associated with more frequent recommended (p=0.005) and implemented changes (p=0.008). Most other factors, including plan complexity and use of stereotactic radiation, were not predictive in this analysis. Comparing the independent versus team PR approach, there was no significant difference in recommended or implemented changes. The mean±standard deviation (SD) staff time required per plan reviewed was 36±6 and 37±6 minutes, including 21±6 and 10±6 minutes of physician time, for team and independent PR, respectively.

Conclusion: This work highlights the high frequency of complex and stereotactic radiation in the palliative setting, along with the importance of timely PR and the potential benefit of reviewing even simple, 2D/3D radiation plans. Additionally, from a process perspective, our work showed that independent PR may require less dedicated physician time.

## Introduction

Quality assurance (QA) programs are an important part of ensuring the best possible clinical care for patients receiving radiation and, a robust QA program includes peer review (PR) of radiation treatment plans. In the context of radiation therapy, PR involves a review of the treatment plan by at least one qualified radiation oncologist (RO) besides the treating physician [1]. This is a crucial step in the treatment planning process as appropriate PR can help to identify plan errors, improve standardization of practice, as well as promote continued education among staff [1-5].

The process of PR can be quite varied between different institutions [6]. It can take a variety of forms, including one-on-one PR, multi-disciplinary team PR, and inter-institutional PR [7]. During one-on-one PR, subsequently referred to as independent PR, a single RO other than the treating physician independently reviews the treatment plan. During multi-disciplinary team PR, subsequently referred to as team PR, professionals from various specialties, such as radiation oncology, medical physics, dosimetry, and radiation therapy, meet to review and discuss radiation plans [7]. For inter-institutional PR, plan review involves various professionals at different institutions [7]. Differences in efficacy and resource utilization between the different PR models are not well understood. 

Much of the literature on PR of radiation treatment plans has focused on curative intent treatment. A systematic review of the literature, which included mainly curative intent treatment plans, estimated that PR led to clinical change in approximately one in nine of all reviewed plans; however, there was a wide range in the rate of clinical change [1]. The data for PR of specifically palliative-intent plans is more limited but also shows notable variation in rates of recommended or implemented changes. The reported rates of recommended changes following palliative PR range from 2.1% to 28% and the rate of implemented changes range from 1.3% to 6.1% [8-11]. Given the scarcity of the literature, we performed a retrospective, single-institution analysis to evaluate the characteristics of reviewed plans as well as the outcomes of PR, including the rate and type of peer feedback along with any factors influencing error identification in palliative-intent radiation plans. Additionally, during the time frame of this study, it transitioned from team PR with weekly meetings to independent PR. This offered the opportunity to compare different PR processes, including the associated time burden of each.

## Materials and methods

Captured data

This single-institution, QA project assessed palliative QA review at a Canadian academic cancer center (London Regional Cancer Program, London, Ontario, Canada), between September 2018 and October 2020. Relevant data were captured by a palliative clinical specialist radiation therapist (pCSRT). Captured data included patients’ age and gender, treating RO, primary diagnosis, treatment site, treatment status, radiation prescription, treatment technique, and additional free-text clinical notes (including prior radiation and systemic therapy treatment details). Data were compiled and retrospectively grouped into standardized categories for 1) diagnosis, 2) treatment site, 3) plan complexity, and 4) the use of stereotactic radiation. Stereotactic radiation was defined during the initial data collection by the pCSRT based on a high dose per fraction. Free-text clinical notes had been captured at the time of the initial treatment plan review by our pCSRT. These were retrospectively reviewed to identify patients with previous radiation and, based on the prior site of treatment, were categorized based on the presence of potential overlap with the treatment plan being peer-reviewed.

Team peer review

From late September 2018 to early March 2020, palliative QA used a team PR approach with in-person rounds, scheduled for one hour each week, and led by the pCSRT with attendance of ROs, medical physicists, as well as RO residents and other learners. Contours, fields, plans, supplemental notes, and images were reviewed by the group, as available. The pCSRT prospectively captured which plans were reviewed and recorded peer feedback, if any, on each plan. Peer feedback was retrospectively categorized as either 1) queries (questions about the plan that were not a specific recommendation) or 2) change recommendations (major or minor). Classification of major versus minor change recommendations was based on definitions by Rouette et al. [12]. A major change was "a change requiring repeat planning and/or having a foreseeable effect on treatment toxicity or cancer outcomes" [12]. A minor change "did not meet the criteria for a 'major' change and did not lead to significant repeat treatment planning" [12]. Categorization was performed retrospectively by two independent reviewers (SG and AM), with discrepancies settled by a third reviewer (GR). Reviewers were blinded to the treating physician and the type of review (independent versus team PR).

Of note, during the team PR period, queries were not recorded if the treating RO was present to address the question during rounds. Attendance of ROs was recorded through a sign-in sheet. This information, along with the frequency of queries recorded when the treating RO was not present, was used to estimate the number of unrecorded queries during team PR. 

Independent peer review

Starting in mid-March 2020 and extending until October 2020, the QA process was changed to independent PR due to the COVID-19 pandemic and the associated need for physical distancing. Initially, familiarity with virtual meeting software was limited, and asynchronous, offline QA was more accessible and more convenient in a time when staffing scarcity was a concern. During this independent PR period, each plan was assigned to a single RO for independent review, as during the team PR period, the pCSRT kept detailed records including the same patient and treatment data as was recorded during team PR along with a record of any peer feedback.

Time estimates

RO attendance and hours spent on PR were determined from weekly attendance sheets, with one pCSRT and one medical physicist present at each meeting. When calculating time per peer feedback, the extrapolated number of queries was used. For the independent PR period, RO PR time was based on the number of plans reviewed and the staff survey estimation of the minutes required to review a palliative plan. Preparation and follow-up time for pCSRT were added based on the self-reported time dedicated to PR per week.

Statistical analysis

Descriptive statistics were generated, stratified by cohort (team versus independent PR), and compared using the chi-square test, Fisher’s exact test, independent two-sample t-test, or Wilcoxon rank sum test, as appropriate. Univariable and multivariable logistic regression were performed for recommend changes and applied changes for all eligible variables for all patients and analyzed per plan. All eligible variables were incorporated into a multivariable regression model and sequentially removed using backward elimination techniques until all remaining covariates had p-values < 0.05 with the exception of the cohort (retained in all models). All statistical analyses were performed using SAS version 9.4 software (SAS Institute, Cary, North Carolina, United States), using two-sided statistical testing at the 0.05 significance level. 

## Results

Reviewed plans

In total, 1942 cases were reviewed, representing 85.7% (1942/2266) of all palliative plans during the study period. Details of the reviewed plans are summarized in Table 1. The unreviewed plans were not included in this study. The most common primaries were lung, breast, and prostate cancer. The most common treatment sites were brain, spine, and bone (non-spinal, non-pelvic). A total of 41.1% (n=799) of the plans were categorized as simple (2D/3D) radiation plans, and 56.0% (n=1087) were complex (volumetric modulated arc therapy (VMAT) or tomotherapy) plans. Approximately one-third (30.4%, n=590) of all plans were stereotactic treatments. Nearly half (47.5%, n=922) of the plans were reviewed before treatment started, 42.9% (n=833) were reviewed while treatment progressed, and 4.2% (n=82) were reviewed after the completion of treatment. Of the 82 plans that were completed prior to PR, the majority (n=54, 65.9%) were single-fraction treatments.

**Table 1:** Baseline characteristics for all reviewed radiation treatment plans during both team and independent PR periods. ^1^For ‘primary diagnosis’ and ‘treated site,’ only categories representing > 2% or > 5%, respectively, of reviewed plans are shown. ^2^Only includes prior radiation that was potentially relevant to the treatment plan being reviewed. SD: standard deviation; VMAT: volumetric modulated arc therapy; PR: peer review

Characteristic	All palliative plans (n=1942)
Gender: n (%)	
Female	930 (47.9)
Male	1012 (52.1)
Age: mean ± SD	67.0 ± 12.0
Radiation treatment status at the time of PR: n (%)	
Before the start of radiation	922 (47.5)
During radiation	833 (42.9)
After the completion of radiation	82 (4.2)
Treatment status missing	105 (5.4)
Primary diagnosis^1^: n (%)	
Lung cancer	645 (33.2)
Breast cancer	253 (13.0)
Prostate cancer	202 (10.4)
Colorectal cancer	108 (5.6)
Renal cell carcinoma	114 (5.9)
Gastrointestinal cancers (excluding colorectal cancer)	103 (5.3)
Melanoma	68 (3.5)
Hepatocellular carcinoma/cholangiocarcinoma	57 (2.9)
Genitourinary (excluding prostate cancer)	53 (2.7)
Sarcoma	43 (2.2)
Treated site^1^: n (%)	
Brain	581 (29.9)
Spine	381 (19.6)
Bone (non-spinal, non-pelvic)	328 (16.9)
Thorax	182 (9.4)
Liver	157 (8.1)
Pelvis (bone)	145 (7.5)
Prescription dose: n (%)	
20 Gy in 5 fractions	649 (33.4)
30 Gy in 5 fractions	187 (9.6)
30 Gy in 10 fractions	181 (9.3)
35 Gy in 5 fractions	157 (8.1)
8 Gy in 1 fraction	141 (7.3)
27 Gy in 3 fractions	90 (4.6)
25 Gy in 5 fractions	72 (3.7)
20 Gy in 1 fraction	64 (3.3)
Other	401 (20.7)
Plan technique: n (%)	
VMAT or tomotherapy	1087 (56.0)
2D/3D	799 (41.1)
Not recorded	56 (2.9)
Previous radiotherapy^2^: n(%)	465 (23.9)
Peer feedback: n (%)	
Major	12 (0.6)
Minor	12 (0.6)
Query	21 (1.1)

Of the 1942 reviewed cases, 1657 (85.3%) underwent team PR, and 285 (14.7%) underwent independent PR. There were significant baseline differences in treatment plan characteristics including diagnosis, treatment site, and plan complexity (see Appendix 1). When transitioning to independent PR, complex plans were prioritized for review. During the independent PR period, 95.8% (n=273) of the reviewed plans were complex and 58.9% (n=168) were stereotactic, compared to 49.1% (n=814) and 25.5% (n=422), respectively, during the team PR period (p < 0.001).

Finally, within team PR only, the presence of the treating RO was also recorded (i.e., was the treating physician present during team PR of their radiation plan?). Over half of the time a plan was reviewed (57.9%), the treating RO was present during the review.

Characteristics and rates of peer feedback

The rate of peer feedback on reviewed plans was 2.3% (45/1942), including 24 recommendations (12 major and 12 minor) and 21 queries. Types of concerns raised during PR were target volume (49%, 22/45), prescribed dose (24%, 11/45), dose to organ-at-risk (OAR) (11%, 5/45), OAR volume (2%, 1/45), appropriateness of treatment plan (7%, 3/45), and other (7%, 3/45). There were 18 implemented changes (0.9%), representing one change per 108 plans reviewed. Two-thirds (67%, 8/12) of major change recommendations were implemented, compared to 25% (3/12) of minor change recommendations and 33% (7/21) of queries (p=0.080).

**Figure 1:**
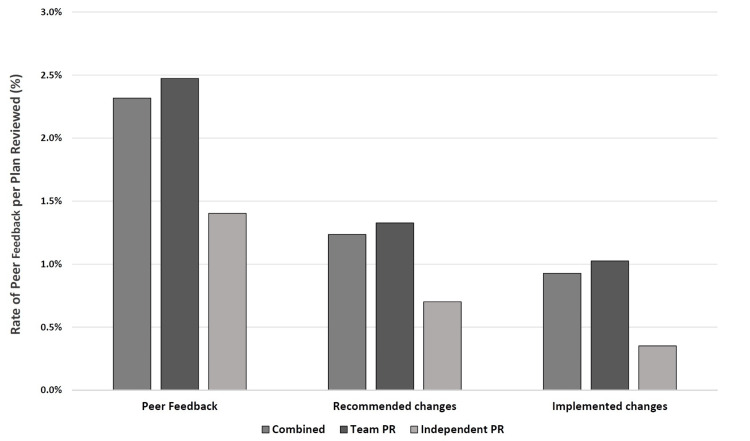
Rate of peer feedback following peer review (PR) over the entire study period (team and independent PR combined) as well as for team and independent PR individually. Peer feedback includes all queries and recommended changes. For team PR, the unrecorded queries were estimated as described.

Comparing team PR and independent PR, the rates of peer feedback were 2.5% (n=41/1657) and 1.4% (n=4/285), respectively (p=0.267). Accounting for the estimated number of unrecorded queries that occurred during team PR (refer to Team peer review in the Materials & Methods section), the rate rose to 3.6% (60/1657), which trended toward significant when compared to independent PR (p=0.053) (Figure 1). Rates of major or minor change recommendations were 1.3% (n=22) and 0.7% (n=2) for team and independent PR, respectively (p=0.145). Rates of implemented changes were 1.0% (n=17) and 0.4% (n=1) and an average of one change was implemented per 97 and 285 plans reviewed for team and independent PR, respectively (p = 0.50).

Predictors of feedback

On multivariable logistic regression, the frequency of recommended changes (major and minor) was impacted by the presence (versus absence) of the treating RO at the time of review (odds ratio (OR): 0.31; 95% confidence interval (CI): 0.12-0.75; p=0.010), and whether the patient had started treatment (OR: 3.80; 95% CI: 1.49-9.67; p=0.005; Table 2). Change recommendations were less likely if the treating RO was present during the plan review, but the rate of implemented change was not affected. Plans that had not yet started were more likely to have recommended and implemented changes (OR: 4.58; 95% CI: 1.49-14.11; p=0.008). Patients with either a breast (OR: 4.33; 95% CI: 1.48-12.69; p=0.008) or prostate diagnosis (OR: 4.64; 95% CI: 1.36-15.76; p=0.014) were more likely to have an implemented change, but the rate of recommended change was not affected.

**Table 2:** Univariable and multivariable logistic regression models for recommended and implemented changes. p-values < 0.05 shown in bold. ^1^Only includes prior radiation that was potentially relevant to the treatment plan being reviewed. OR: odds ratio; CI: confidence interval; PR: peer review; NR: not reported; VMAT: volumetric modulated arc therapy

	Recommended change		Implemented change	
	OR (95% CI)	p-value	OR (95% CI)	p-value
Univariable:				
Independent vs. team PR	0.53 (0.12, 2.25)	0.385	0.34 (0.05, 2.56)	0.295
Male vs. female	0.56 (0.24, 1.28)	0.168	0.60 (0.23, 1.55)	0.289
Age (per 5 years)	0.99 (0.84, 1.17)	0.909	0.90 (0.75, 1.07)	0.212
Treating radiation oncologist present (vs. no)	0.33 (0.14, 0.82)	0.017	0.51 (0.19, 1.33)	0.167
Radiation treatment started (vs. no)	3.51 (1.39, 8.90)	0.008	4.15 (1.36, 12.66)	0.013
Primary diagnosis (vs. no)				
Lung cancer	1.69 (0.75, 3.79)	0.204	0.39 (0.11, 1.35)	0.138
Breast cancer	0.58 (0.14, 2.50)	0.466	3.24 (1.20, 8.74)	0.020
Prostate cancer	0.78 (0.18, 3.35)	0.740	2.50 (0.81, 7.66)	0.110
Renal cell carcinoma	1.51 (0.35, 6.49)	0.584	2.10 (0.48, 9.27)	0.327
Melanoma	1.29 (0.17, 9.77)	0.803	NR	NR
Hepatocellular carcinoma/cholangiocarcinoma	1.50 (0.20, 11.30)	0.696	NR	NR
Genitourinary cancers (excluding prostate cancer)	1.55 (0.21, 11.69)	0.672	NR	NR
Sarcoma	4.05 (0.92, 17.84)	0.064	2.54 (0.33, 19.53)	0.372
Treated site (vs. no)				
Brain	1.46 (0.64, 3.37)	0.373	0.70 (0.23, 2.13)	0.529
Spine	0.56 (0.17, 1.89)	0.352	1.51 (0.53, 4.27)	0.438
Bone (non-spinal, non-pelvic)	0.97 (0.33, 2.87)	0.960	1.88 (0.66, 5.30)	0.235
Thorax	0.40 (0.05, 2.98)	0.371	0.53 (0.07, 4.03)	0.542
Liver	1.72 (0.51, 5.85)	0.386	0.71 (0.09, 5.41)	0.743
Pelvis (bone)	1.09 (0.25, 4.69)	0.908	1.48 (0.34, 6.52)	0.603
Lower extremity	1.01 (0.13, 7.62)	> 0.99	NR	NR
Gastrointestinal	1.08 (0.14, 8.12)	0.941	1.50 (0.20, 11.46)	0.695
Shoulder	3.39 (0.77, 14.81)	0.106	NR	NR
Stereotactic radiation (vs. no)	1.56 (0.67, 3.65)	0.305	2.19 (0.85, 5.65)	0.104
Plan technique: VMAT/tomotherapy (vs. 2D/3D)	1.32 (0.55, 3.13)	0.981	1.64 (0.60, 4.53)	0.975
Previous radiotherapy^1^ (vs. no)	0.84 (0.31, 2.25)	0.723	1.23 (0.44, 3.47)	0.697
Multivariable:				
Independent vs. team PR	0.26 (0.06, 1.15)	0.075	0.34 (0.05, 2.60)	0.300
Treating radiation oncologist present (vs. no)	0.31 (0.12, 0.75)	0.010	--	--
Radiation treatment started (vs. no)	3.80 (1.49, 9.67)	0.005	4.58 (1.49, 14.11)	0.008
Primary diagnosis (vs. no)				
Breast cancer	--	--	4.33 (1.48, 12.69)	0.008
Prostate cancer	--	--	4.64 (1.36, 15.76)	0.014

Rate of feedback of PR was similar between simple, 2D/3D versus VMAT or tomotherapy radiation plans as well as between stereotactic versus non-stereotactic radiation plans with no significant difference in the rate of recommended or implemented change (Table 2). Other examined factors, including the type of plan review (team versus independent PR), demographics, site, and previous radiation, were also not significantly predictive of recommended or implemented changes. Primary diagnoses of breast or prostate cancer were both associated with higher rates of implemented, but not recommended changes. Additionally, as a sensitivity analysis, univariable logistic regression was repeated for team PR plans only to examine the effect of the number of ROs present. The frequency of recommended or implemented changes was not significantly affected by the number of ROs present during PR (see Appendix 2).

Time dedicated to palliative PR

The mean number of ROs present during the weekly team PR meeting was 8.1±2.4 (range: 2-14). The mean weekly total time requirement of all oncology staff to participate in team PR for palliative plans was estimated at 14.1±2.4 hours per week, including 8.1±2.4 hours of total physician time per week. The mean time per plan reviewed was 35.7±6.0 minutes. The estimated mean time per independent PR plan reviewed was similar to team PR at 37.1±6.4 minutes; however, the burden on physician time was less (20.5±6.0 minutes versus 9.7±6.4 minutes per plan reviewed for team versus independent PR). Further examination of the effect of the number of ROs present as well as the effect of differing rates of recommended or implemented change reported in prior studies is further examined in Appendix 3.

## Discussion

Our study provides a detailed retrospective examination of palliative PR at a large academic center. We report on relevant demographic data, including the high frequency of complex and stereotactic treatment techniques used in the palliative setting. Additionally, we present an analysis of predictors of recommended and implemented changes in palliative radiation treatment following PR along with a novel comparison of the peer feedback and time required for two different PR approaches.

Comparing our data to the available literature, the rate of peer feedback for team PR of palliative plans was similar to that found by Thompson et al., who reported a rate of recommended and implemented changes of 2.1% (29/1413) and 1.3% (19/1413), respectively, following team PR [8]. Other publications have reported higher rates of peer feedback in palliative plans. Mitchell et al. reported a rate of implemented changes of 6.1% (7/115) and Qureshi et al. reported a 15% (3/20) rate of recommended changes following team PR, albeit both on very small samples [9,11]. Another publication, Walburn et al. reported rates of peer feedback for a large number of plans that underwent both ‘early’ team PR within 48 hours of CT simulation as well as ‘late’ team PR after treatment planning was completed [10]. They also found a high rate of recommended changes at 28% (143/508) [10]. The wide variation in the rates of recommended and implemented changes is also seen in the curative-intent PR literature. A pooled analysis of PR of 11,491 predominantly radical plans showed a weighted mean change rate of 10.8%, with a wide variance (3.0%-100%) across different series [1].

Interpreting rates of peer feedback can be challenging and we lack a good benchmark metric for the rate of radiation plan peer feedback, especially in the palliative setting. The striking ranges may be attributable to multiple factors, including a lack of formalized and consistent change definitions, inconsistencies in recording recommendations, group culture regarding peer critique, relative consistency in clinical practice between oncologists, different PR models, and retrospective data collection. Despite the high importance placed on palliative PR in our institution, the relatively low peer feedback rate observed in our cohort may have been influenced by any of these factors. Additionally, not all peer feedback necessarily leads to a change in treatment and institutional practice can vary on how this is handled. Within our own institution, the implementation of queries or recommended changes is left to the discretion of the treating physician.

Reasons behind why recommendations were not implemented were not consistently tracked within our retrospective data; however, we did find that major recommendations were more likely to be implemented compared to minor recommendations or queries. Within our analysis, we used the definition of major versus minor recommendation suggested by Rouette et al. [12]. In their work, a major change was “a change requiring repeat planning and/or having a foreseeable effect on treatment toxicity or cancer outcomes”, while a minor change “did not meet the criteria for a 'major' change and did not lead to significant repeat treatment planning” [12]. They did not use queries within their work, which we defined here as questions about the plan that were not a specific recommendation. There is a lack of standardization within the literature of definitions of these categories and we suggest that the definitions we use here are a reasonable starting point. Standardization of definitions, along with prospective tracking of recommendations and treating physician’s responses to peer feedback with well-defined categorization across institutions would help to improve comparability and overall PR quality moving forward.

The time required for PR is a commonly cited barrier to broader implementation [13]. As such, analyzing and optimizing process efficiency is of interest, especially in the setting of growing stresses on healthcare resources. Independent PR required approximately half as much physician time per plan reviewed as team PR (20.5±6.0 minutes and 9.7±6.4 minutes, respectively). Additionally, our study did not find a significant difference in peer feedback rates between independent PR when compared to team PR. While there may have been a trend toward a higher rate of peer feedback when including queries, there was no difference in recommended or implemented changes. This suggests that while a team-based approach may generate more discussion, this may not necessarily translate into actual changes in the treatment plan. Additionally, subsequent analysis within the team PR model did not identify a clear relationship between the number of ROs present and the likelihood of recommended or implemented changes. However, given that we cannot know the ideal or optimal rate of peer feedback, we cannot truly comment on the comparative efficacy of team PR versus independent PR.

Nevertheless, taken together, these findings raise the potential for future work looking at the optimal number of physicians involved in team PR as well as further comparisons of the outcome of team versus independent PR, ideally in a prospective manner and with a large sample. An Independent review of identical plans using different PR processes would allow a better assessment of the efficacy of each method. Additionally, as a potential aspect of this future work, we note that the rapid acceleration of artificial intelligence in radiation oncology may also represent an exciting horizon to increase the efficacy and throughput of PR processes, with the potential for augmentation or replacement in some settings [14]. However, the lack of defined guidelines, subjective target volumes, and complexity in cases of re-irradiation will require careful evaluation of large language models to assess efficacy. Further, while striving for efficiency remains crucial, we must also recognize the less measurable benefits of PR that may not be met as well by certain PR processes such as independent PR, including inter-disciplinary discussions, learning for trainees, and continued education for physicians.

In evaluating outcomes of PR, our analysis identified several predictors for peer feedback on multivariable logistic regression. The timing of PR was found to be a significant predictor, with radiation plans reviewed prior to treatment initiation having significantly higher rates of peer feedback. This is consistent with prior work in the non-palliative setting [10,15]. Early PR provides a better opportunity to address concerns and adapt the treatment plan. However, palliative treatments tend to have fewer fractions and start quickly to manage symptoms, making PR prior to the start of treatment difficult and decreasing the opportunity for change. While many institutions, including ours, meet on a weekly basis for team PR [6], more frequent PR could decrease this problem. Thompson et al. routinely reviewed palliative plans twice a week with additional ad-hoc sessions scheduled to review urgent plans, and, using this approach, 96.4% of plans were reviewed before or on the day of the start of treatment [8]. Alternatively, a hybrid model, wherein urgent cases are reviewed by independent PR after approval by the treating physician as opposed to waiting for weekly team PR, may reduce the number of cases being reviewed after the start of treatment, while minimizing the additional time burden on physicians of the frequent PR required to ensure cases are reviewed prior to the start of treatment.

The finding that the absence of the treating RO was predictive of a change recommendation can be interpreted in different ways. It is possible that their presence dissuaded criticism, or that they provided rationale, negating the need for formal change recommendation. The presence of the treating RO did not significantly affect the rate of implemented changes, which supports the latter interpretation. However, sample size may also play a role in this given that there were more recommended changes than implemented changes. Nevertheless, we also note that in the relatively anonymized independent PR model, the rate of recommendations did not increase. This decreases the likelihood that ‘social intimidation’ plays a significant role, but is clouded by the potential lack of rigor in a single plan reviewer. Verification would require a prospective, narrative review.

Interestingly, neither plan complexity nor stereotactic technique was found to be predictive of peer feedback, suggesting potential value in reviewing even 2D/3D radiation plans. This is consistent with prior work by Walburn et al., where the radiation treatment technique (3D-CRT (conformal radiation therapy) versus IMRT (intensity-modulated radiation therapy) versus SBRT (stereotactic body radiation therapy) versus electron versus other) was not found to be a significant predictor of change recommendation (p = 0.71) [10]. Given the potential impact on institutional PR practice, this would be worth further exploration within a larger data set. However, it should also be acknowledged that while the similar rate of peer feedback for stereotactic versus non-stereotactic plans could reflect a true similarity in the rate of issues, it does also raise the possibility that there was not always sufficient expertise in general palliative team PR when reviewing more specialized cases. Given the rising complexity of palliative radiation and the increasing proportion of stereotactic treatments [16,17], it is important to consider the expertise present for palliative PR. One change our institution has made in our PR process subsequent to this study is that brain stereotactic cases are now reviewed in a separate set of specialized PR rounds.

Finally, we note that primary cancer types, specifically breast and prostate, were identified as statistically significant predictors of a completed change in the treatment plan. The clinical implications and rationale for this are unclear. This is potentially attributable to small sample size, unrecognized confounders, practice patterns of treating physicians in those sites, or the comparatively favorable prognosis of these disease sites warranting a lower threshold for implementing potentially meaningful alterations. 

The findings of our work must be considered with respect to its limitations. This is a single-institution study with a limited sample size. The relatively small sample size of the independent PR period represents an important limitation of this analysis. The study is also subject to the inherent biases that may be present in retrospective studies. Being a retrospective review, the collection of peer feedback also represents a limitation. As stated, queries were not recorded if they were addressed by the treating physician during team PR. To address this, we calculated an estimate of these ‘unrecorded’ queries, which is subject to error (refer to Peer review in the Materials & Methods section). In contrast to queries, recommendations were always recorded, and based on our analysis, the absence of the treating physician was associated with a higher rate of recommendation. Therefore, the method used to account for ‘unrecorded’ queries could potentially have led to an overestimation of the ‘unrecorded’ queries. If this is the case, then the true rate of queries should lie in between the adjusted and unadjusted rate (i.e., between 2.5% and 3.6%). A prospective, narrative evaluation would help to further characterize these issues.

Nevertheless, despite the methodologic limitations, this analysis provides novel, hypothesis-generating data on an important topic. Palliative radiotherapy is increasing in complexity with more conformal treatments, increasing rates of re-treatment, and increasing the utilization of radiation therapy. While the rate of change was low, major changes were recommended in varying plan complexities, indications, and treatment modalities. Having the right individuals in the right environment at the right time will improve plan quality, departmental collaboration, and learning. Continued efforts to define the ‘right’ mix, and to optimize the PR process for all radiation plans are needed to ensure value for all stakeholders. Ideally, PR models should be compared in a prospective, randomized manner to help reduce bias and subjectivity inherent in the published works to date.

## Conclusions

QA of palliative radiation remains an understudied area of the literature. Our work demonstrates the high frequency of complex and stereotactic radiation in the palliative setting and examines predictors of peer feedback within the setting of palliative QA along with the potential benefit of reviewing even simple, 2D/3D radiation plans. Timely PR, before the start of radiation is strongly predictive of peer feedback; however, as seen in our work, this can be challenging for palliative radiation. Further work looking into palliative QA, especially into comparisons of different QA processes, may help to ensure timely PR while still seeking to minimize the time burden of QA on physicians. Our work showed that independent PR can be less burdensome in terms of physician time, but additional study is needed comparing team versus independent PR processes, especially in terms of the rigor and efficacy of peer review.

## Appendices

Appendix 1

**Table 3:** Baseline characteristics for all reviewed radiation treatment plans by type of peer review (PR) (team PR versus independent PR). ^1^For ‘primary diagnosis’ and ‘treated site’ only, categories representing > 2% or > 5%, respectively, of reviewed plans are shown. ^2^Only includes prior radiation that was potentially relevant to the treatment plan being reviewed. SD: standard deviation; VMAT: volumetric modulated arc therapy

Characteristic	N	Team PR (n=1657)	Independent PR (n=285)	p-value
Gender: n (%)	1942			0.025
Female		811 (48.9)	119 (41.8)	
Male		846 (51.1)	166 (58.3)	
Age: mean ± SD	1942	67.1 ± 12.1	66.9 ± 11.0	0.809
Treating radiation oncologist present: n (%)	1657	960 (57.9)	--	--
Number of radiation oncologists present: mean ± SD	1657	8.09 ± 2.38	--	--
Number of radiation oncologists present: n (%)	1657			
≤ 4		101 (6.1)	--	--
5		131 (7.9)	--	--
6		170 (10.3)	--	--
7		312 (18.8)	--	--
8		290 (17.5)	--	--
9		129 (7.8)	--	--
≥ 10		524 (31.6)	--	--
Radiation treatment status at time of PR: n (%)	1942			< 0.001
Before the start of treatment		753 (45.4)	169 (59.3)	
During radiation		721 (43.5)	112 (39.3)	
After completion of radiation		79 (4.8)	3 (1.1)	
Treatment status missing		104 (6.3)	1 (0.4)	
Primary diagnosis^1^: n(%)	1942			
Lung cancer		563 (34.0)	82 (28.8)	0.085
Breast cancer		231 (13.9)	22 (7.7)	0.004
Prostate cancer		172 (10.4)	30 (10.5)	0.941
Colorectal cancer		79 (4.8)	29 (10.2)	< 0.001
Renal cell carcinoma		92 (5.6)	22 (7.7)	0.151
Gastrointestinal (excluding colorectal cancer)		75 (4.5)	28 (9.8)	< 0.001
Melanoma		49 (3.0)	19 (6.7)	0.002
Hepatocellular carcinoma/cholangiocarcinoma		45 (2.7)	12 (4.2)	0.167
Genitourinary cancers (excluding prostate)		46 (2.8)	7 (2.5)	0.759
Sarcoma		39 (2.4)	4 (1.4)	0.314
Treated site^1^: n (%)	1942			
Brain		470 (28.4)	111 (39.0)	< 0.001
Spine		346 (20.9)	35 (12.3)	< 0.001
Bone		286 (17.3)	42 (14.7)	0.294
Thorax		169 (10.2)	13 (4.6)	0.003
Liver		121 (7.3)	36 (12.6)	0.002
Pelvis (bone)		133 (8.0)	12 (4.2)	0.024
Gastrointestinal		60 (3.6)	19 (6.7)	0.016
Pelvis (soft-tissue)		38 (2.3)	15 (5.3)	0.005
Prescribed dose: n (%)	1942			< 0.001
20 Gy in 5 fractions		612 (36.9)	37 (13.0)	
30 Gy in 5 fractions		140 (8.5)	47 (16.5)	
30 Gy in 10 fractions		170 (10.3)	11 (3.9)	
35 Gy in 5 fractions		113 (6.8)	44 (15.4)	
8 Gy in 1 fraction		138 (8.3)	3 (1.1)	
27 Gy in 3 fractions		52 (3.1)	38 (13.3)	
25 Gy in 5 fractions		51 (3.1)	21 (7.4)	
20 Gy in 1 fraction		44 (2.7)	20 (7.0)	
Other		337 (20.3)	64 (22.5)	
Plan technique: n (%)	1942			< 0.001
VMAT/tomotherapy		814 (49.1)	273 (95.8)	
2D/3D		793 (47.9)	6 (2.1)	
Not recorded		50 (3.0)	6 (2.1)	
Previous radiotherapy^2^: n (%)	1942	395 (23.8)	70 (24.6)	0.792

Appendix 2

**Table 4:** Univariable logistic regression of the effect of the number of treating radiation oncologists present during team peer review (PR) on rates of recommended and implemented changes. OR: odds ratio; CI: confidence interval; NR: not reported

	Recommended change		Implemented change	
	OR (95% CI)	p-value	OR (95% CI)	p-value
Univariable:				
Number of radiation oncologists present (per 1 radiation oncologist)	0.99 (0.83, 1.19)	0.936	1.05 (0.86, 1.28)	0.642
≥ 3 radiation oncologists present	NR	NR	NR	NR
≥ 4 radiation oncologists present	NR	NR	NR	NR
≥ 5 radiation oncologists present	NR	NR	NR	NR
≥ 6 radiation oncologists present	1.64 (0.38, 7.05)	0.508	1.22 (0.28, 5.38)	0.790
≥ 7 radiation oncologists present	2.04 (0.60, 6.95)	0.252	1.50 (0.43, 5.25)	0.525
≥ 8 radiation oncologists present	1.10 (0.47, 2.58)	0.835	0.85 (0.33, 2.22)	0.740

Appendix 3

**Table 5:** Effect of the number of radiation oncologists (ROs) presenting during weekly team peer review (PR) as well as differing rates of recommended and implemented changes on the time burden of palliative PR. Time estimates are provided based on the mean ± standard deviation (SD) as well as the minimum and maximum number of ROs present at team PR during the study period. ^1^Calculated using estimate to account for unrecorded queries during team PR as described. ^2^Calculations account for the two sequential episodes of team PR based on the process described in Walburn et al. [10].

	Mean ± SD	Minimum	Maximum
Number of ROs present during team PR	8.1 ± 2.4	2	14
Time per week (hours)	14.1 ± 2.4	8.0	20.0
Time per plan reviewed (minutes)	35.7 ± 6.0	20.3	50.7
Time per peer feedback^1^ (hours)	16.4 ± 2.8	9.3	23.3
Time per major/minor change recommendation (hours)	44.8 ± 7.6	25.5	63.6
Time per recommended change (hours) – Thompson et al. [8]	28.3 ± 4.8	16.1	40.2
Time per recommended change (hours) – Qureshi et al. [11]	4.0 ± 0.7	2.3	5.6
Time per recommended change (hours) – Walburn et al. [10]^2^	4.2 ± 0.7	2.4	6.0
Time per implemented change (hours)	58.0 ± 9.8	32.9	82.4
Time per implemented change (hours) – Thompson et al. [8]	45.8 ± 7.7	26.0	65.0
Time per implemented change (hours) – Mitchell et al. [9]	9.8 ± 1.6	5.5	13.9
